# Tubularized urethral reconstruction using everted saphenous vein graft in a beagle model

**DOI:** 10.1186/s12894-021-00833-4

**Published:** 2021-04-17

**Authors:** Dan Li, Zhou Shen, Yujie Xu

**Affiliations:** 1 Department of General Practice, The First Affiliated Hospital of Wannan Medical College (Yijishan Hospital of Wannan Medical College), Wuhu, 241000 China; 2 Department of Urology, Anhui Provincial Hospital, Anhui Medical University, Hefei, Anhui China; 3 Department of Urology, The First Affiliated Hospital of Wannan Medical College (Yijishan Hospital of Wannan Medical College), 2 Zheshan West Road, Wuhu, 241000 Anhui Province China

**Keywords:** Urethral stricture, Saphenous vein graft, Urethral reconstruction, Tubularized urethroplasty

## Abstract

**Background:**

A long segment stricture in the anterior urethra is a challenge in urology. We conducted a study to investigate the efficacy of anterior urethral reconstruction using an everted saphenous vein graft (SVG) in a tubular fashion.

**Methods:**

Twelve male beagles were randomly divided into three groups: experimental group (n = 5), control group (n = 5) and normal group (n = 2). A 3 cm defect in the anterior urethra was created. Autologous SVG was harvested. In the experimental group, urethral defect was replaced by an everted SVG in a tubular fashion. In the control group, urethral reconstruction was performed using an uneverted SVG. Beagles in all groups received retrograde urethrography to evaluate urethral patency and were killed for histological examination 6 months after operation.

**Results:**

Four beagles in the experimental group had no voiding difficulty and the other one could not void spontaneously. Retrograde urethrography showed the four beagles in experimental group had wide urethral lumens. Ether urethral stricture or fistula were detected in all animals in the control group. Histological analysis of the four beagles in the experimental group indicated the everted SVG completely integrated into the urethra. The reconstructed urethra contained a wide lumen and was completely covered by urothelium. The periurethral collagen and muscle fibers formed and were highly organized. Everted SVG showed a high ability of neovascularization. In the control group, the reconstructed segment showed a fibrotic urethral lumen where the urothelium was not intact. Only few new capillaries were formed.

**Conclusions:**

Everted SVG demonstrates for a promising strategy for potential urethral stricture repair.

## Background

Adult and pediatric disorders of the urethra including hypospadias, trauma, and stricture require substituted urethroplasty to preserve the function of the urinary tract. Reconstruction of the anterior urethra is one of the most challenging problems in urology [1].

Autologous tissues have advantages of excellent biological compatibility as well as rapid and effective neovascularization. Oral mucosa and penile skin flap are preferred substituted materials for urethral reconstruction [2, 3]. Onlay urethroplasty and multi-stage repair are commonly used techniques [4]. In some cases, these methods have problems including stricture recurrence, fistula formation and inadequate donor material. Harvesting oral mucosa is associated with donor site morbidity, such as submucosal scarring, pain, numbness and injury to salivary ducts [5, 6]. These issues highlight a critical need for the development of alternative material and reconstructive strategies for anterior urethral repair.

The saphenous vein is commonly used as vascular substitute material. It is easily harvested, and its associated wound complications were not dramatic, including chronic pain, numbnes and paresthesia/dysesthesia [7]. We previously performed urethroplasty in a rabbit model using everted SVG as an onlay graft and gained encouraging results [8]. The inner wall of blood vessels consist of vascular endothelium. We hypothesized that everting vein graft makes the outside vascular endothelium cling to periurethral tissue. In turn, the endothelium quickly attracts blood supply from the surrounding periurethral tissues including dartos. Here, we compare differences in neovascularization between everted SVG and non-everted SVG.

In addition, saphenous vein has a similar caliber as the urethra in humans. Even though tubularized grafts have not shown to be a preferential form of substitution, it avoids multi-stage surgery of ventral and dorsal onlay urethroplasty. Whether everted SVG can be used for tubularized urethraplasty remains unkown. Thus, we performed anterior urethral reconstruction in a beagle model using everted SVG in the tubular form.

## Methods

### Urethroplasty using everted SVG

This experimental animal protocol was approved by the Animal Experimentation Ethics Committee of Wannan Medical College in accordance with the Guide for the Care and Use of Laboratory Animals. Twelve male beagle dogs (Animal Research Center of Wannan Medical College. Wuhu, China) weighing 8–10 kg were randomly divided into three groups: experimental group (n = 5), control group (n = 5) and normal group (n = 2).

**Fig. 1:**
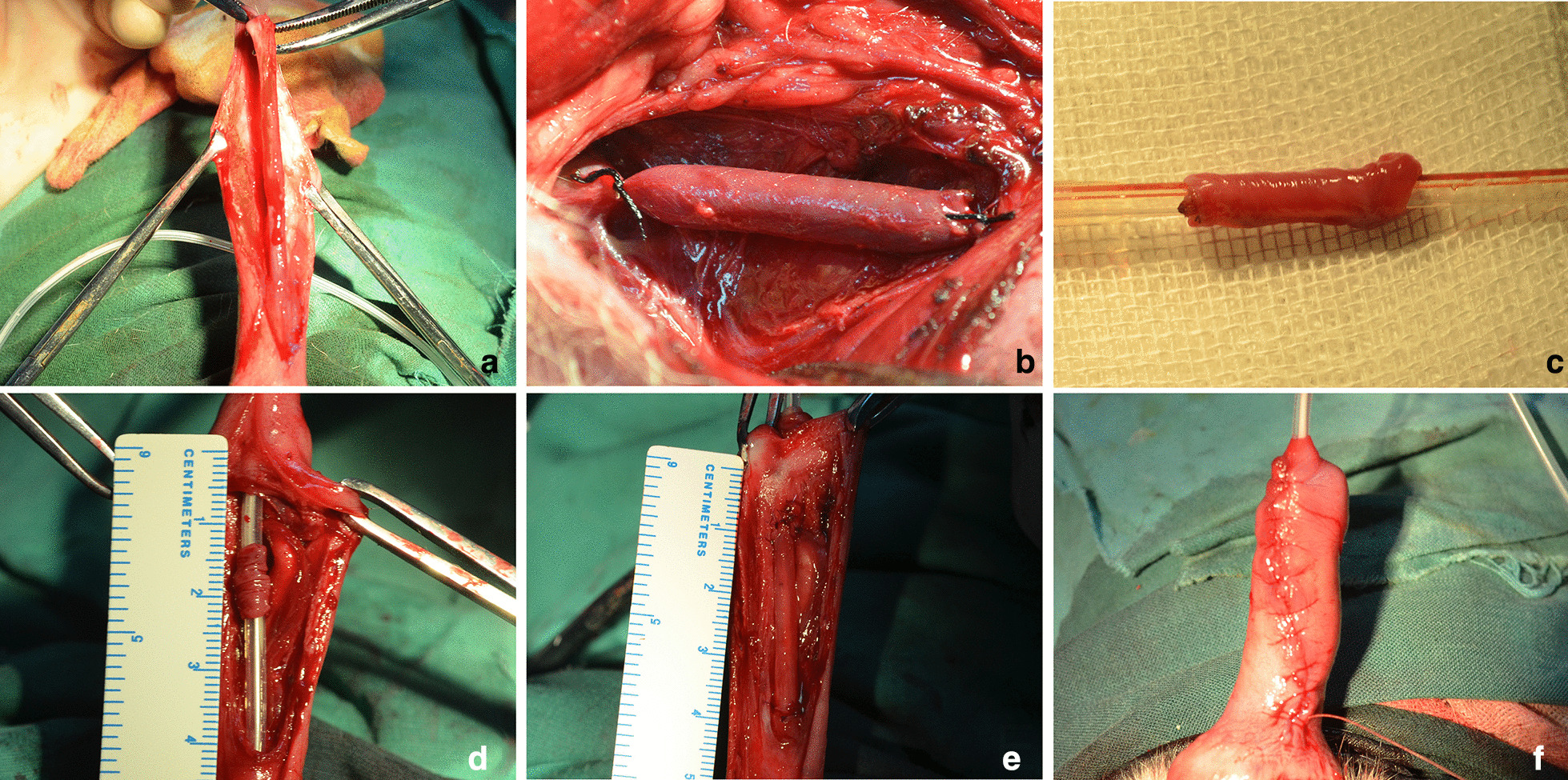
Anterior urethral reconstruction with SVG. **a** A segment of anterior urethra was separated. **b** A 3-cm SVG was harvested. **c** The SVG was everted in experimental group. **d** The urethral catheter was inserted as a stent. **e** The urethral defect was repaired by SVG in tubular fashion. **f** The subdermal layer in penis and the skin was closed

After general anesthesia with 3% pentobarbital, the beagles in the experimental group and control group were placed on an operating table in supine position. The right thigh and genital organ were shaved and prepared with povidone-iodine solution. A 4-cm incision was performed on the ventral skin of penis. A 3 cm segment of anterior urethra was removed between the external urethral orifice and bulbar urethra, in order to make a urethral defect (Fig. 1a).

A 4-cm incision was carried out on the right thigh skin and a 3-cm SVG was excised (Fig. 1b). In the experimental group, the vein grafts were everted like taking off sleeves,without longitudinally cutting the venous wall, (in order to make the endothelium outside) (Fig. 1c). A 8 F catheter was inserted into the urethra and was used as a stent (Fig. 1d). Urethral defect was repaired by the everted SVG in tubular fashion with 6–0 vicryl separated sutures (Fig. 1e). In the control group, the urethral defect was repaired using uneverted SVG in the same fashion. Nonabsorbable sutures were placed at the anastomosis margins for mark. Muscular tissue around the reconstructed urethra and the skin incision in penis was closed in layers (Fig. 1f). In the normal group, no surgical intervention was performed. The procedures for all animals were performed under sterile conditions by the same surgeon.

### Postoperative care

An Elizabetan collar was placed around the neck of animal to prevent the catheter removal. The catheter was left indwelling for two weeks. All animals in the experimental group and control group received penicillin 100U/kg/day intramuscularly for 14 days.

### Retrograde urethrography

Retrograde urethrography was performed every month after operation. After general anesthesia, the animals were placed obliquely on an examination table. A Foley (6-F) catheter was inserted into the urethra until the balloon entered the urethral orifice. Inflate the balloon with 1 ml sterilized water. X-ray images of urethra were obtained under the fluoroscopy after contrast agent was gently injected through the catheter.

### Histological and immunohistochemical analysis

Animals were euthanized by an overdose of 3% pentobarbital and air embolization 6 months after operation. The penises were obtained and the segments of the reconstructed urethra were extracted within the area outlined by the marking sutures. These segments were placed in 10% formalin, dehydrated in graded alcohols, and then embedded in paraffin. Sections (5 mm) were cut and then stained with hematoxylin–eosin (HE) staining, Masson’s trichrome(MT) staining, and immunohistochemical(IHC) staining using CD-31 and uroplakin-II(UP-II) antibodies.

IHC staining using CD-31 antibody were quantified to compare neovascularization of the grafts using ImageJ software, in 10 different fields for each tissue sample. Data for these measurements are shown as mean ± SD. Statistical analysis was performed using a t-test through SPSS®, with *p* < 0.05 was considered as statistically significant.

Radiologists and pathologists were blinded to the group when they were evaluating.

## Results

The surgical procedures were successfully performed on all beagles. There were no major intraoperative complications observed. During the 6 months follow-up period, four beagles in the experimental group showed no voiding difficulty and the other one could not spontaneously void. Two beagles in control group could not spontaneously void and they underwent cystostomy with a suprapubic catheter. Urogenic cutaneous fistula in penis was detected in the other three animals in control group.

### Retrograde urethrography

**Fig. 2:**
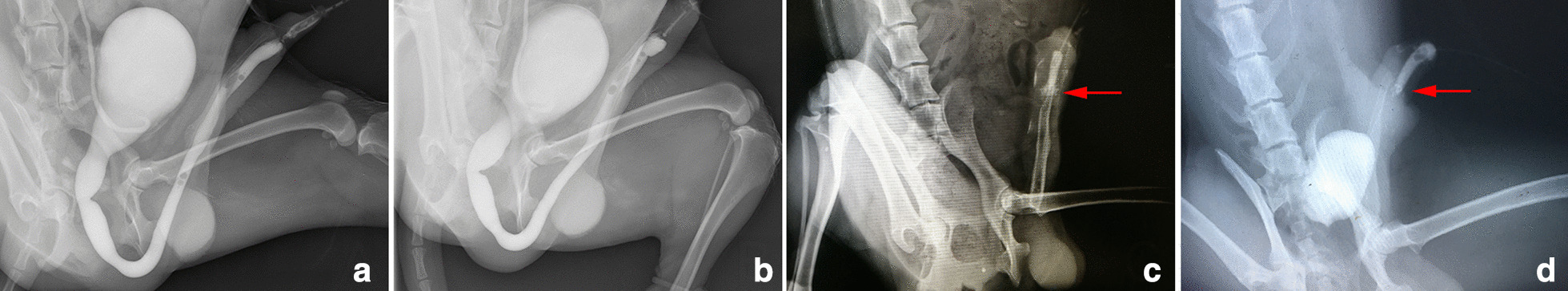
Retrograde urethrography. **a** Urethrogram in beagle in normal group. **b** Urethrogram in experimental group 6 months postoperatively. **c** Urethral fistula in control group (red arrow point to the contrast leak). **d** Urethral stricture in control group (red arrow point to the stricture)

The retrograde urethrograms showed the urethral caliber of four beagles in the experimental group were similar to that of normal beagles (Fig. 2a, b). The contrast agent freely passed through the urethra without any signs of stricture or contrast leak. These indicate that the four beagles in the experimental group maintained wide urethral lumen 6 months postoperatively. The one beagle in the experimental group could not void spontaneously developed urethral stenosis. Ether significant urethral stricture or fistula were detected in all animals in control group (Fig. 2c, d).

### Histological and IHC examination

**Fig. 3:**
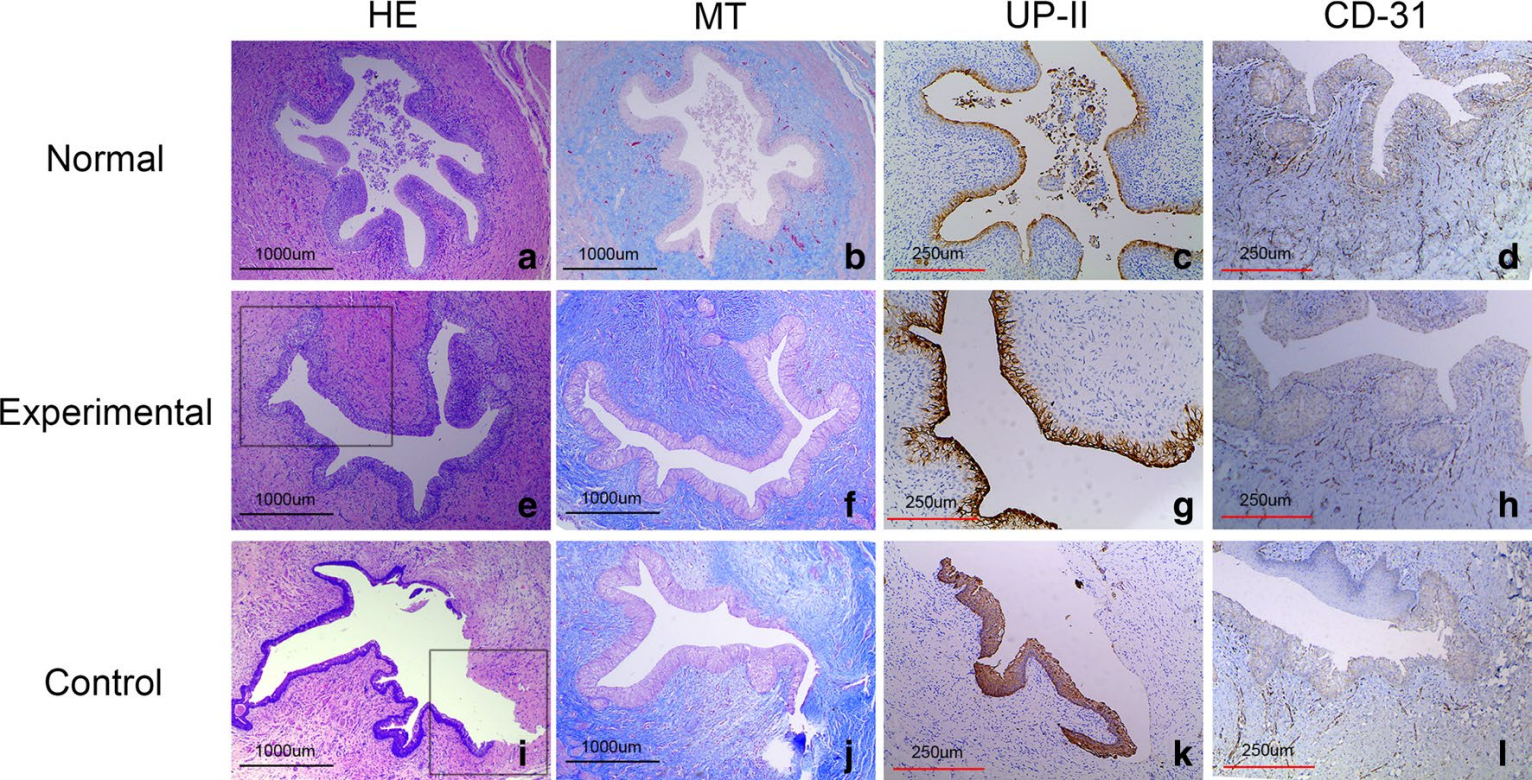
Histological and IHC analysis. **a**–**d** HE staining, MT staining, IHC staining with UP-II and CD31 antibodies of the reconstructed urethra in normal group. **e** Wide urethral lumen was completely covered by endothelium in experimental group. **f** MT staining showed highly organized collagen fibers and muscle tissue in subepithelium. **g** IHC staining with UP-II antibody showed expression of uroplakin in the endothelium. **h** IHC staining with the CD31 showed dense capillaries in subepithelium. **i** HE staining showed a narrow urethral lumen and urothelium defect in control group. **j** MT staining showed fibrosis with abundant collagen. **k** IHC staining with the UP-II revealed a fragment of urothelium. **l** Vascular capillaries were sparse in control group

In the experimental group, HE staining indicated the urethral lumen of the reconstructed segment was completely covered by the urothelium (Fig. 3e). There was no obvious difference observed in the normal urethra (Fig. 3a). MT staining showed highly organized collagen fibers and muscle tissue in the reconstructed urethra, but the muscle tissue seemed to be less than what was present in the normal urethra (Fig. 3b, f). The UP-II antibody is a special urothelium marker. IHC staining with UP-II antibody showed positive expression in the urethral inner wall (Fig. 3g).

In the control group, the reconstructed segment showed a narrow urethral lumen and the urothelium was not intact (Fig. 3i). MT staining showed abundant collagen with strong blue staining, indicating severe fibrosis 6 months postoperatively (Fig. 3j). IHC staining with UP-II antibody revealed a fragment of urothelium in the urethral inner wall (Fig. 3k).

**Fig. 4:**
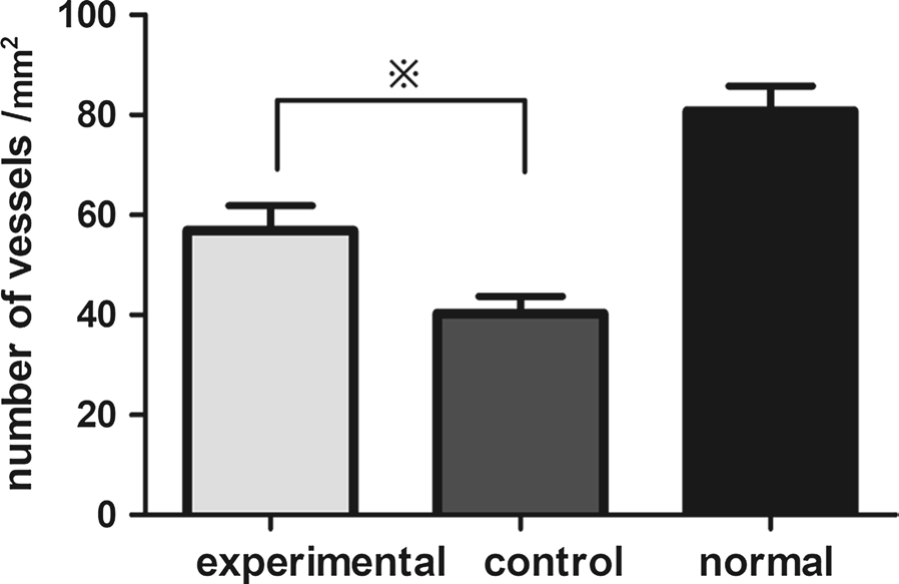
Histomorphometric analysis of the extent of CD31 + vessels present in the 3 groups. ※Signifificant difference among the experimental and control groups (*P* < .01)

IHC staining using a CD31 antibody was performed to observe the neovascularization of implanted tissue. In the experimental group, IHC staining with a CD31 antibody showed newly formed dense capillaries after 6 months (Fig. 3h). In the control group, IHC staining using a CD31 antibody indicated sparse vascular capillaries (Fig. 3l). Histomorphometric analysis found that the number of newly formed capillaries in the experimental group was higher than the control group (56.90 ± 1.57 vessels/mm^2^ vs 40.30 ± 1.06 vessels/mm^2^, *p* < 0.01) (Fig. 4).

## Discussion

Various urethral conditions often require substitute urethral reconstruction including long-segment urethral strictures, traumatic defects, complicated hypospadias and previous failed urethroplasty [9]. Several substituted materials have been adopted for clinical urethroplasty, including full-thickness skin graft, bladder mucosa, oral mucosa (buccal or lingual mucosa) and colonic mucosa [10–12]. Buccal mucosa and preputial skin graft remain the widely used tissues for urethral replacement. However, there are associated complications, such as graft necrosis, hair growth, stricture recurrence, and fistula formation [13, 14]. In addition, harvesting buccal mucosa may also lead to possible morbidities such as intraoperative hemorrhage, submucosal scarring, pain, postoperative infection, and injury to salivary ducts [3, 15]. Wood et al. [16] reported the morbidity of buccal mucosa grafts harvested for urethroplasty in 57 patients. Of these patients, 68% had perioral numbness that persisted after 6 months in 26% of the patients, 83% developed postoperative pain, 67% initially had difficulty with mouth opening that persisted after 6 months in 9% of the patients, and 2% had a mucous retention cyst. When urethral defects are complex, donor tissue extracted from oral cavity or prepuce may be insufficient. For these reasons, many attempts have been made to select alternative tissues that can serve as adequate urethral substitutes. Tissue engineering may be a promising option for the generation of an artificial urethra. However, accompanied techniques are complex containing multi-steps and the results of preliminary clinical applications are not satisfactory [17, 18]. Up to now, tissue engineered urethra is mainly being performed in animal experiments and has not made its way to the clinic [19].

Tuffer et al*.* performed urethral reconstruction using vein graft for the first time in 1910 [14]. Subsequently, several animal trials have been reported using vein grafts for urethral reconstruction [20–22]. Kahveci et al*. *[20] suggested that a vein graft mainly acts as a scaffold and the endothelium sloughs off after 3 days. Hubner et al. [22] and Foroutan et al. [23] used everted vein grafts and showed improved outcomes. They believed their modified technique eliminated negative effects on urine stream from the valve inside the venous lumen. We previously performed urethroplasty in a rabbit model with saphenous vein patch as onlay graft and gained encouraging results [8]. Results of everted vs non-everted vein grafts showed more optimal results with the everted graft.

To the best of our knowledge, one of the key factors in reconstructing urethra is adequate blood supply and fast neovascularization of the implanted graft [24, 25]. Insufficient blood supply inevitably results in shrinkage of grafts and the formation of fibrosis. In the study, the saphenous vein was everted and the vascular endothelium became outside. We hypothesized that everting the vein graft making the vascular endothelium on the outside facilitates the everted SVG blood supply gain from surrounding periurethral tissues including dartos. In this study, IHC staining with CD31 antibody showed dense vascular capillaries in the experimental group. Histomorphometric analysis demonstrated that compared to noneverted SVG, everting the vein graft improved its ability of neovascularization.

There is a lack of studies investigating one-stage reconstruction using a tubularized graft. Even though tubularized grafts have not proven to be a preferential substitution, they avoid multi-stage surgery of ventral combined dorsal onlay urethroplasty. The saphenous vein is a hollow tubular structure with a similar diameter of the urethra. The saphenous vein is readily accessible, even for the most extensive urethral reconstruction. Adult male beagles were selected as our experimental model because the urinary tract in beagle resemble the urinary tract in human. Foroutan et al. [23] performed histological study after euthanizing rabbits 7, 10, 14, 22, and 30 days after operation, they found that gradual uroepithelialization occurred within one month and demonstrated that the vein graft functioned as a guide for uroepithelium migration. The histological examination in our previous experiment revealed similar results [8]. The everted vein graft showed full integration in the neourethra. Urethral lumen was completely covered by regenerative uroepithelium 6 months postoperatively. Fistula formation and stenosis at the anastomosis were noted in the animals of control group. The experimental group showed improvement over the control group both in retrograde urethrography and histologic analysis. The MT staining showed that well-formed collagen and muscle fibers in the experimental group. However, abundant collagen fiber, narrow urethral lumen and urothelium defect were observed in control group. The rapid survival of the implant graft contributed to its function, serving as a barrier against urine extravasation, as well as facilitating urothelium cell migration and proliferation in the newly formed urethral tissue [26].

In this study, we create urethral defect models in the healthy urethra of normal animals and cannot fully resemble the exact clinical situation of urethral stricture in humans, which is characterized by the fibrotic urethra bed. Other limitations of this study include a small sample size and a short follow-up time. Further investigations with longer follow-up time are necessary to assess its technical applicability and to translate this technology into clinic.

## Conclusion

We first used the everted SVG for tubularized urethral reconstruction in a beagle model. In our study, 80% of the experimental beagles showed no voiding difficulty or urethral fistula. The animals showed wide urethral lumen during the observation period. Everted SVG demonstrates promise for anterior urethral stricture repair. This study provides preclinical evidence and information needed to move toward clinical studies of this technique.

## Data Availability

The datasets used and/or analysed during the current study available from the corresponding author on reasonable request.

## Abbreviations

SVG: Saphenous vein graft
HE: Hematoxylin–eosin
MT: Masson’s trichrome
IHC: Immunohistochemical
UP-II: Uroplakin-II
